# Association between glycated hemoglobin A1c and diabetic retinopathy in adults with type 2 diabetes mellitus residing at high altitudes

**DOI:** 10.3389/fendo.2026.1889928

**Published:** 2026-08-27

**Authors:** Yanjun Chen, Bin Zhang, Yuanzheng Liu, Haijing Wang, Li Tong, Guanghua Xi, Wen Peng, Weirong Pu

**Affiliations:** 1 Department of Traditional chinese Medicine, Medical College, Qinghai University, Xining, China; 2 School of Mathematics and Statistics, Qinghai Minzu University, Xining, China; 3 Department of Public Health, Medical College, Qinghai University, Xining, China; 4 Qinghai Provincial Key Laboratory of Prevention and Control of Glucolipid Metabolic Diseases with Traditional Chinese Medicine, Xining, China; 5 Department of Endocrinology, Qinghai Provincial Hospital of Traditional Chinese Medicine, Xining, China

**Keywords:** altitude, diabetic retinopathy, effect modification, glycated hemoglobin A1c, type 2 diabetes mellitus

## Abstract

**Aims:**

To investigate the association between glycated hemoglobin A1c (HbA1c) and diabetic retinopathy (DR) in adults with type 2 diabetes mellitus (T2DM) at high altitude, and to explore potential effect modifiers.

**Methods:**

This retrospective, hospital-based, cross-sectional study included 1040 adults with T2DM residing at high altitude in Qinghai Province, stratified into two altitude groups (1800–2500 m vs. ≥2500 m); no low-altitude comparison group was included. DR was diagnosed by fundus photography. Logistic regression, restricted cubic spline analyses, stratified and interaction analyses were performed.

**Results:**

DR prevalence was 26.6% (n = 277). After full adjustment, each 1% increase in HbA1c was associated with higher odds of prevalent DR (OR 1.19, 95% CI 1.12-1.25), with no evidence of nonlinearity. Altitude did not significantly modify the association (*P* for interaction = 0.213). Significant effect modification was observed for estimated glomerular filtration rate (eGFR) (*P* for interaction = 0.031) and a borderline interaction was observed for body mass index (BMI) (*P* for interaction = 0.061). The association strengthened with lower eGFR (*P* for trend = 0.016) and higher BMI (*P* for trend = 0.013), being strongest in the low eGFR plus high BMI group (OR 1.38, 95% CI 1.20-1.59).

**Conclusions:**

Among high-altitude adults with T2DM, HbA1c exhibited a linear positive association with prevalent DR; lower eGFR significantly modified this association, whereas the modifying trend linked to higher BMI remains exploratory and needs additional confirmation. These findings suggest that glycemic control, weight status, and renal function may need to be considered jointly when assessing DR burden in high-altitude populations.

## 1 Introduction

Diabetic retinopathy (DR) is a vision-threatening microvascular complication of diabetes. DR is associated with all-cause mortality and the occurrence of cardiovascular events in populations with diabetes (1). Besides diabetes duration, factors associated with retinopathy include chronic hyperglycemia (2), nephropathy (3), hypertension (4), and dyslipidemia (5). Glycated hemoglobin A1c (HbA1c), reflecting average glycemia over the preceding 2–3 months, is a core predictive biomarker for DR among populations living at sea level or low altitudes (6).

High-altitude hypoxia induces a series of systemic physiological adaptations and metabolic alterations, including hematological adaptations (7), glucose homeostasis reprogramming (8), fluctuations in renal function (9), changes in body mass index (BMI) (10), and retinal microcirculatory abnormalities (11). These alterations may collectively influence HbA1c levels as well as the onset and progression of DR. Recent study has demonstrated that chronic hypoxia not only stimulates erythropoiesis and cellular glucose uptake but also redirects glucose metabolism toward the Luebering-Rapoport (LR) shunt and glycolytic intermediates, thereby facilitating oxygen release from hemoglobin (Hb), and improving glucose tolerance (8). Moreover, erythrocyte count was an independent determinant of elevated HbA1c levels (12), while erythrocytosis has been associated with retinal microcirculatory dysfunction, thickening of the retinal nerve fiber layer, and retinal venous dilation (11, 13). Furthermore, higher hemoglobin levels have been associated with a lower prevalence of DR among individuals living at high altitude (14).

Beyond hematological alterations, hypoxia itself may induce pathological retinal changes, including increased retinal blood flow, disruption of the blood-retinal barrier, and enhanced oxidative stress (15). Elevated HbA1c can further aggravate retinal hypoxia, thereby accelerating the development of DR (16). In addition, reduced estimated glomerular filtration rate (eGFR) implies impaired renal clearance and the accumulation of circulating pro-inflammatory and pro-angiogenic mediators that may exacerbate retinal barrier disruption (17). The association between BMI and DR remains controversial, with positive, null, and even inverse associations reported (18, 19), possibly due to population heterogeneity.

Accordingly, the role of HbA1c in DR under high-altitude hypoxic conditions remains incompletely understood. Prior small-scale Tibetan cohort (n=239) reported a non-significant independent association between HbA1c and DR (20), while epidemiological evidence demonstrates progressive dissociation between HbA1c and ambient glucose concentrations as elevation rises (21). The landmark Diabetes Control and Complications Trial (DCCT) demonstrated a strong relationship between HbA1c levels and DR risk, based on the premise that lower levels of glycemia would reduce the development and/or progression of complications and the recognition of HbA1c as a marker of long-term average glycemia (6). This altitude-induced decoupling may challenge the core prerequisite of the DCCT glucose hypothesis: HbA1c can no longer faithfully reflect true glycemic burden, which may distort its predictive relationship with DR. Together, these findings raise uncertainty as to whether the positive HbA1c-DR association persists among high-altitude residents with type 2 diabetes mellitus (T2DM) and whether altitude and other clinical characteristics amplify or attenuate this association.

Large-scale stratified interaction analyses investigating the HbA1c-DR relationship in high-altitude populations with T2DM remain scarce. No prior work has simultaneously evaluated the dual modifying effects of BMI and eGFR. To address this critical gap, we conducted a cross-sectional study to examine the association between HbA1c and DR in adults with T2DM at high altitude. We further explored whether this association varied across subgroups of clinical characteristics, which could provide insights into minimizing diabetic complications in this unique setting. Finally, we predefined two primary hypotheses: I. HbA1c exhibits an independent positive linear association with DR prevalence among adults with T2DM living at high altitudes. II. Among high-altitude T2DM participants, residential altitude does not modify the magnitude of the HbA1c-DR association; eGFR and BMI act as independent effect modifiers of this relationship.

## 2 Methods

### 2.1 Study participants

**Figure 1:**
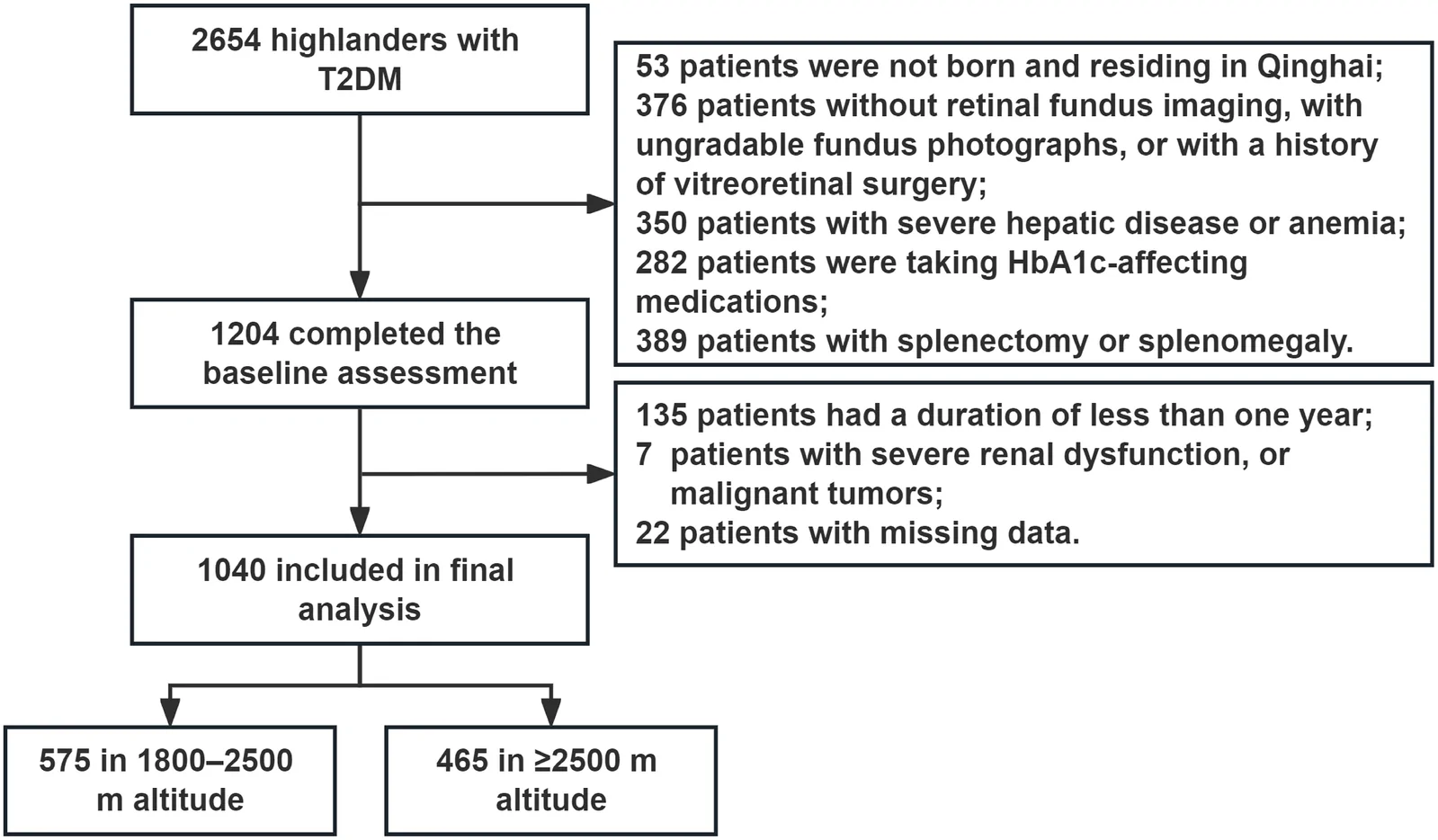
Flowchart of study participant selection.

This retrospective, hospital-based, cross-sectional study screened 2654 consecutive adults with T2DM admitted to the Department of Endocrinology between January 2024 and January 2025. Individuals who met all of the following inclusion criteria were included: 1) completed standardized retinal fundus imaging without a history of prior vitreoretinal surgery; 2) had fully available laboratory data for renal function and HbA1c quantification; 3) had medical records documenting Qinghai Province as their birthplace and long-term place of residence; 4) were over 18 years of age. Exclusion criteria were as follows: 1) Anemia, defined as hemoglobin levels <120 g/L in males and <110 g/L in females, which alters erythrocyte lifespan and distorts HbA1c measurements; 2) kidney failure (chronic kidney disease [CKD] stage 5), defined as eGFR <15 mL/min/1.73 m² for at least three months, which confounds both glycemic biomarker stability and microvascular complications; 3) severe hepatic disease or malignant neoplasms, which interfere with systemic metabolism, glycemic biomarker measurements and retinal vascular homeostasis; 4) a history of splenectomy or clinically confirmed splenomegaly, conditions that disrupt erythrocyte turnover and distort HbA1c readouts; 5) current use of medications known to interfere with HbA1c interpretation or erythrocyte turnover, including erythropoietin, iron supplements, and high-dose systemic corticosteroids; 6) T2DM duration less than 1 year, newly diagnosed patients may have unstable diagnostic status, enter glycemic remission after early intensive hypoglycemic or lifestyle interventions, and lack sufficient cumulative hyperglycemic exposure; 7) incomplete data. A detailed participant flow diagram is presented in **Figure 1**.

The final sample comprised 1040 participants stratified by residential altitude (1800–2500 m and ≥2500 m). Residential altitude was estimated based on county-level government seat altitude from the 2024 Qinghai Statistical Yearbook (22), which was used as a proxy measure of individual residential altitude because precise residential geolocation and lifetime altitude exposure data were unavailable from the medical records. This 2500 m cutoff was selected based on well-established physiological and epidemiological evidence. According to the international consensus on chronic high-altitude diseases, 2500 m has been set as the threshold for high-altitude illnesses (23). In addition, a nationwide cross-sectional study confirmed that the association between altitude and HbA1c markedly increases above 2500 m (24), indicating altered HbA1c validity at higher elevations. The lower boundary of 1800 m was determined by the minimum elevation of county-level government seats in Qinghai Province and was used to define the study population rather than to represent a physiological threshold.

The study was approved by the Medical Ethics Committee of Qinghai Provincial Hospital of Traditional Chinese Medicine (protocol QZYEC20250425-077; April 27, 2025) and conducted according to the Declaration of Helsinki. Written informed consent was waived due to the retrospective design. All data were anonymized.

Sample size: Because this was a retrospective hospital-based study, no formal *a priori* sample size calculation was performed. The final analytic sample included 1,040 participants, including 277 patients with DR, providing an adequate number of outcome events for the primary association analysis. *Post-hoc* power calculations indicated the study had 84% power to detect an odds ratio of 1.19 for the association between HbA1c (per 1% increase) and DR at α = 0.05 in the overall study population. However, some subgroup analyses with small cell sizes, particularly ethnicity-stratified analyses, may have been underpowered and should be interpreted as exploratory.

### 2.2 Measurements

All demographic, clinical, laboratory, and ophthalmic data were obtained during the same hospitalization. Demographic and clinical data included age, sex, ethnicity, duration of T2DM, glucose-lowering medication use, smoking status, BMI, systolic blood pressure (SBP), diastolic blood pressure (DBP), and hypertension history. Hypertension was defined as SBP ≥140 mmHg and/or DBP ≥90 mmHg on three occasions or use of antihypertensive drugs.

Blood samples were collected after an overnight fast of at least 8 h, and all laboratory tests were performed in the Department of Laboratory Medicine of our hospital. Routine blood tests were conducted using an automated hematology analyzer (BC-7500, Mindray, Shenzhen, China). Fasting lipid profiles including triglycerides (TG), total cholesterol (TC), high-density lipoprotein cholesterol (HDL-C), and low-density lipoprotein cholesterol (LDL-C), serum creatinine (Scr), and oral glucose tolerance test (OGTT) glucose levels were measured using an automated clinical chemistry analyzer (AU5800, Beckman Coulter, Brea, CA, USA). HbA1c was measured using a latex-enhanced immunoturbidimetric assay with HbA1c reagents (Medicalsystem Biotechnology, Ningbo, China) on an automated clinical chemistry analyzer (AU5800, Beckman Coulter, Brea, CA, USA), according to the manufacturer’s instructions. The reagent package insert specifies compatibility with the AU5800 analytical platform. Routine internal quality control was performed by the hospital laboratory, which operates under ISO 15189-accredited quality management procedures.

eGFR was calculated using the 2009 Chronic Kidney Disease Epidemiology Collaboration (CKD-EPI) creatinine equation based on serum creatinine, age, and sex. The area under the OGTT blood glucose curve (AUC_G_) was calculated using the trapezoidal rule from blood glucose measurements at 0, 0.5, 1, 2, and 3 hours. Specifically, AUC_G_ was calculated as the sum of the areas of adjacent trapezoids: [(G0 + G0.5)/2 × 0.5] + [(G0.5 + G1)/2 × 0.5] + [(G1 + G2)/2 × 1] + [(G2 + G3)/2 × 1], where G0, G0.5, G1, G2, and G3 represent glucose concentrations at 0, 0.5, 1, 2, and 3 h, respectively.

All participants underwent routine ophthalmology consultation during hospitalization. After pharmacological pupil dilation with compound tropicamide eye drops, color fundus photographs were obtained using a conventional 45° non-mydriatic retinal camera (CR6-45NM; Canon, Japan). In addition, visual acuity testing, intraocular pressure measurement, and slit-lamp examination were routinely performed. Fundus photographs were evaluated by an ophthalmologist as part of routine clinical care. The ophthalmologist was unaware of participants’ HbA1c levels and other laboratory results at the time of image interpretation and provided an ophthalmology consultation diagnosis together with treatment recommendations. The ophthalmology consultation records and fundus photographs were subsequently reviewed by an attending physician in the Department of Endocrinology and incorporated into the final inpatient diagnosis.

DR was diagnosed and graded according to the Evidence-based guidelines for diagnosis and treatment of diabetic retinopathy in China (2022) (25), based on the presence of microaneurysms, hemorrhages, hard exudates, cotton-wool spots, intraretinal microvascular abnormalities, venous beading, neovascularization, vitreous or preretinal hemorrhage, and fibrosis. Fundus photographs judged as ungradable because the fundus could not be adequately visualized (“fundus not clearly visible” in the ophthalmology consultation report) were excluded from the study during participant selection. Proliferative DR was rare and not analyzed separately.

### 2.3 Statistical analysis

Complete-case analysis was performed; all participants with incomplete covariate or outcome data were excluded from the analytical cohort. The Shapiro-Wilk test was used to assess the normality of continuous variables. Because continuous variables were non-normally distributed, they were summarized as median (interquartile range) and compared using the Mann–Whitney U test. Categorical variables were presented as n (%) and compared using the chi-square test.

Logistic regression models were used to evaluate the association between HbA1c and diabetic retinopathy (DR), and results were reported as odds ratios (ORs) with 95% confidence intervals (CIs). Three hierarchical models were constructed. Model 1 was the unadjusted crude model. Model 2 was adjusted for demographic and metabolic covariates, including age, sex, ethnicity, diabetes duration, BMI, glucose-lowering medication use, smoking status, hypertension, and LDL-C. Model 3 was the primary fully adjusted model, further adjusted for renal function and hematological covariates, including eGFR and Hb. Covariates were selected *a priori* based on previous literature and their potential clinical relevance as confounders of the association between HbA1c and DR. Residential altitude was not included in the primary regression models because its modifying effect was evaluated through prespecified stratified and interaction analyses. For subgroup, trend, and sensitivity analyses, residential altitude was additionally included as a continuous covariate to further control for residual altitude-related confounding.

Restricted cubic spline (RCS) regression was used to assess the potential nonlinear association between HbA1c and DR. Four knots were selected based on commonly used recommendations in epidemiological studies to provide sufficient flexibility for modeling nonlinear associations while minimizing the risk of overfitting. The overall association was assessed using the likelihood ratio test (LRT), and nonlinearity was evaluated by testing the nonlinear spline components. Statistical significance for nonlinear associations was defined as *P* for nonlinearity <0.05.

Stratified analyses were performed by age (< 60 vs. ≥ 60 years), sex (Men vs. Women), ethnicity (Han, Tibetan, and Other minorities), duration (< 10 vs. ≥ 10 years), glucose-lowering medication use (never/intermittent vs. continuous), BMI (< 24 vs. ≥ 24 kg/m²), smoking status (never vs. former/current), hypertension (present vs. absent), LDL-C (< 2.6 vs. ≥ 2.6 mmol/L), eGFR (<105 vs. ≥ 105 mL/min/1.73 m²; the cutoff was selected as the midpoint of the normal eGFR range [90–120 mL/min/1.73 m²] to distinguish relatively lower versus higher eGFR levels within the normal range), Hb (< 162 vs. ≥ 162 g/L), and red blood cell (RBC) count (< 5.3 vs. ≥ 5.3×10¹²/L). Effect modification by binary stratification variables was assessed by adding multiplicative interaction terms between HbA1c and each stratification variable to the multivariable logistic regression model, and *P* values for interaction were obtained using the Wald test. For the multi-category ethnicity variable, global interaction was assessed using the likelihood ratio test by comparing models with and without the HbA1c ×ethnicity interaction terms. Subgroup and interaction analyses were exploratory in nature, and no formal adjustment for multiple testing was applied.

To further evaluate whether the association between HbA1c and DR varied across different levels of BMI and eGFR, BMI and eGFR were categorized into quartiles. BMI quartiles were defined as Q1 (<22.77), Q2 (22.77–24.82), Q3 (24.82–27.17), and Q4 (≥27.17 kg/m²); eGFR quartiles were defined as Q1 (<96.37), Q2 (96.37–103.68), Q3 (103.68–112.18), and Q4 (≥112.18 mL/min/1.73 m²). Linear trends across ordered quartile strata were tested by assigning integer scores from 1 to 4 to the quartile categories and modeling these scores as continuous variables in interaction terms with HbA1c.

A combined eGFR-BMI stratified analysis was also performed by cross-classifying participants according to median eGFR (<103.68 vs. ≥103.68 mL/min/1.73 m²) and median BMI (<24.82 vs. ≥24.82 kg/m²). The statistical interaction between eGFR and BMI was assessed using the LRT.

Sensitivity analyses were performed by modeling residential altitude as a continuous variable and by redefining altitude strata using alternative cutoffs of 2400 m and 2600 m. Additionally, contemporaneous glucose measurements (fasting plasma glucose [FPG], 2-hour postprandial glucose [2hPG], and AUC_G_) were further included in the fully adjusted model to evaluate the robustness of the observed HbA1c-DR association independent of current glycemic status. All statistical tests were two-sided; *P* < 0.05 was significant. Analyses were performed using R software (version 4.5.2; R Foundation for Statistical Computing, Vienna, Austria).

## 3 Results

### 3.1 Baseline characteristics

Among 1040 participants, median age was 58.0 (51.0-63.0) years, 299 (28.8%) were women, and median duration was 8.0 (4.0-13.0) years. DR prevalence was 26.6%. The distribution of DR severity among study participants is summarized in **Supplementary Table 1**. Compared with the 1800–2500 m group, participants at ≥2500 m were younger, with a higher proportion of Tibetan ethnicity, elevated BMI, LDL-C, eGFR, and RBC counts, alongside 2hPG and AUC_G_ (all *P* < 0.05). However, HbA1c did not differ significantly between altitude strata (*P* = 0.323). The prevalence of DR was 27.7% in the 1800–2500 m group and 25.4% in the ≥2500 m group, with no statistically significant difference between the two groups (*P* = 0.450) (**Table 1**). Baseline characteristics of included participants and excluded representative samples are compared in **Supplementary Table 2**.

**Table 1:** Baseline demographic and clinical characteristics of the study participants.

Characteristics at baseline	Total	1800–2500 m	≥2500 m	*P*-value^a^
No	1040	575	465	
Age,years	58.0 (51.0, 63.0)	58.0 (52.0, 65.0)	56.0 (50.0, 62.0)	<0.001
Women, n (%)	299 (28.8)	160 (27.8)	139 (29.9)	0.507
Ethnicity, n(%)				<0.001
Han	617 (59.3)	397 (69.0)	220 (47.3)	
Tibetan	228 (21.9)	72 (12.5)	156 (33.6)	
Other minorities	195 (18.8)	106 (18.4)	89 (19.1)	
Duration, years	8.0 (4.0, 13.0)	8.0 (4.0, 13.5)	8.0 (4.0, 13.0)	0.304
Non-continuous medication use, n (%)	359 (34.5)	193 (33.6)	166 (35.7)	0.513
BMI, kg/m^2^	24.82 (22.77, 27.17)	24.49 (22.49, 26.67)	25.35 (23.38, 27.68)	<0.001
Hypertension, n(%)	397 (38.2)	225 (39.1)	172 (37.0)	0.521
SBP, mmHg	127.0 (115.0, 140.0)	128.0 (116.0, 140.0)	126.0 (113.0, 139.0)	0.085
DBP, mmHg	78.0 (71.0, 86.0)	78.0 (71.0, 87.0)	77.0 (70.0, 85.0)	0.239
Smoker, n(%)	246 (23.7)	136 (23.7)	110 (23.7)	1.000
DR, n (%)	277 (26.6)	159 (27.7)	118 (25.4)	0.450
HbA1c, %	7.9 (6.5, 9.7)	7.8 (6.5, 9.6)	8.0 (6.5, 9.7)	0.323
FPG, mmol/L	7.30 (5.96, 9.19)	7.39 (5.97, 9.40)	7.20 (5.95, 9.01)	0.182
2hPG, mmol/L	14.50 (11.43, 17.49)	14.77 (11.69, 17.92)	14.14 (11.10, 16.94)	0.013
AUC_G_, mmol·h/L	37.03 (29.50, 44.87)	37.47 (30.06, 45.33)	36.08 (28.84, 43.27)	0.029
TG, mmol/L	1.85 (1.26, 2.83)	1.84 (1.23, 2.86)	1.85 (1.31, 2.82)	0.693
HDL-C, mmol/L	1.07 (0.93, 1.27)	1.08 (0.93, 1.29)	1.07 (0.91, 1.24)	0.165
LDL-C, mmol/L	2.60 (2.17, 3.15)	2.58 (2.12, 3.11)	2.67 (2.21, 3.21)	0.040
TC, mmol/L	4.64 (4.00, 5.40)	4.61 (3.97, 5.39)	4.68 (4.05, 5.43)	0.237
Scr, μmol/L	58.50 (50.27, 68.30)	59.20 (50.45, 68.75)	57.60 (50.20, 67.50)	0.255
eGFR, mL/min/1.73 m²	103.68 (96.37, 112.18)	102.72 (95.92, 110.51)	104.60 (97.32, 113.54)	0.006
RBC, ×10^12^/L	5.30 (4.94, 5.69)	5.27 (4.94, 5.62)	5.36 (4.98, 5.77)	0.042
Hb, g/L	162.0 (150.0, 173.0)	161.0 (150.0, 171.5)	163.0 (150.0, 175.0)	0.098

a Between altitude 1800–2500 m and ≥2500 m.

SBP, systolic blood pressure; DBP, diastolic blood pressure; DR, diabetic retinopathy; FPG, fasting plasma glucose; 2hPG, 2-hour postprandial glucose; AUC_G_, area under the glucose curve; TG, triglycerides; HDL-C, high-density lipoprotein cholesterol; LDL-C, low-density lipoprotein cholesterol; TC, total cholesterol; Scr, serum creatinine; eGFR, estimated glomerular filtration rate; RBC, red blood cell count; Hb, haemoglobin.

### 3.2 Association between HbA1c and DR prevalence

In univariate analyses (**Table 2**), older age, longer duration of T2DM, hypertension, higher SBP, HDL-C, Scr, HbA1c, and glucose measures (FPG, 2hPG, AUC_G_) were positively associated with DR. In contrast, eGFR and non-continuous medication use (defined as complete absence of hypoglycemic medication or intentional long-term intermittent drug discontinuation due to poor adherence, excluding occasional single missed doses) were inversely associated, indicating protective associations in the unadjusted analyses (all *P* < 0.05).

**Table 2:** Unadjusted associations between clinical characteristics and diabetic retinopathy.

Variables	OR (95% CI)	*P*-value
Age	1.05 (1.03, 1.06)	<0.001
Women	0.94 (0.69, 1.27)	0.683
Tibetan	0.78 (0.54, 1.10)	0.161
Other minorities	0.80 (0.54, 1.15)	0.228
Duration	1.17 (1.14, 1.20)	<0.001
Non-Continuous medication use	0.57 (0.42, 0.77)	<0.001
Altitude	1.00 (1.00, 1.00)	0.328
BMI	0.99 (0.95, 1.03)	0.512
Hypertension	2.18 (1.65, 2.89)	<0.001
SBP	1.01 (1.01, 1.02)	<0.001
DBP	1.01 (0.99, 1.02)	0.356
Smoker	0.81 (0.58, 1.12)	0.215
HbA1c	1.14 (1.09, 1.20)	<0.001
FPG	1.06 (1.01, 1.10)	0.018
2hPG	1.07 (1.04, 1.11)	<0.001
AUC_G_	1.02 (1.01, 1.03)	0.001
TG	0.97 (0.90, 1.03)	0.338
HDL-C	2.11 (1.31, 3.42)	0.002
LDL-C	1.11 (0.93, 1.33)	0.230
TC	1.08 (0.96, 1.21)	0.186
Scr	1.01 (1.00, 1.02)	0.003
eGFR	0.98 (0.97, 0.99)	<0.001
RBC	0.90 (0.70, 1.14)	0.382
Hb	1.00 (0.99, 1.00)	0.302

OR, odds ratio; CI, confidence interval; SBP, systolic blood pressure; DBP, diastolic blood pressure; FPG, fasting plasma glucose; 2hPG, 2-hour postprandial glucose; AUC_G_, area under the glucose curve; TG, triglycerides; HDL-C, high-density lipoprotein cholesterol; LDL-C, low-density lipoprotein cholesterol; TC, total cholesterol; Scr, serum creatinine; eGFR, estimated glomerular filtration rate; RBC, red blood cell count; Hb, haemoglobin.

**Figure 2:**
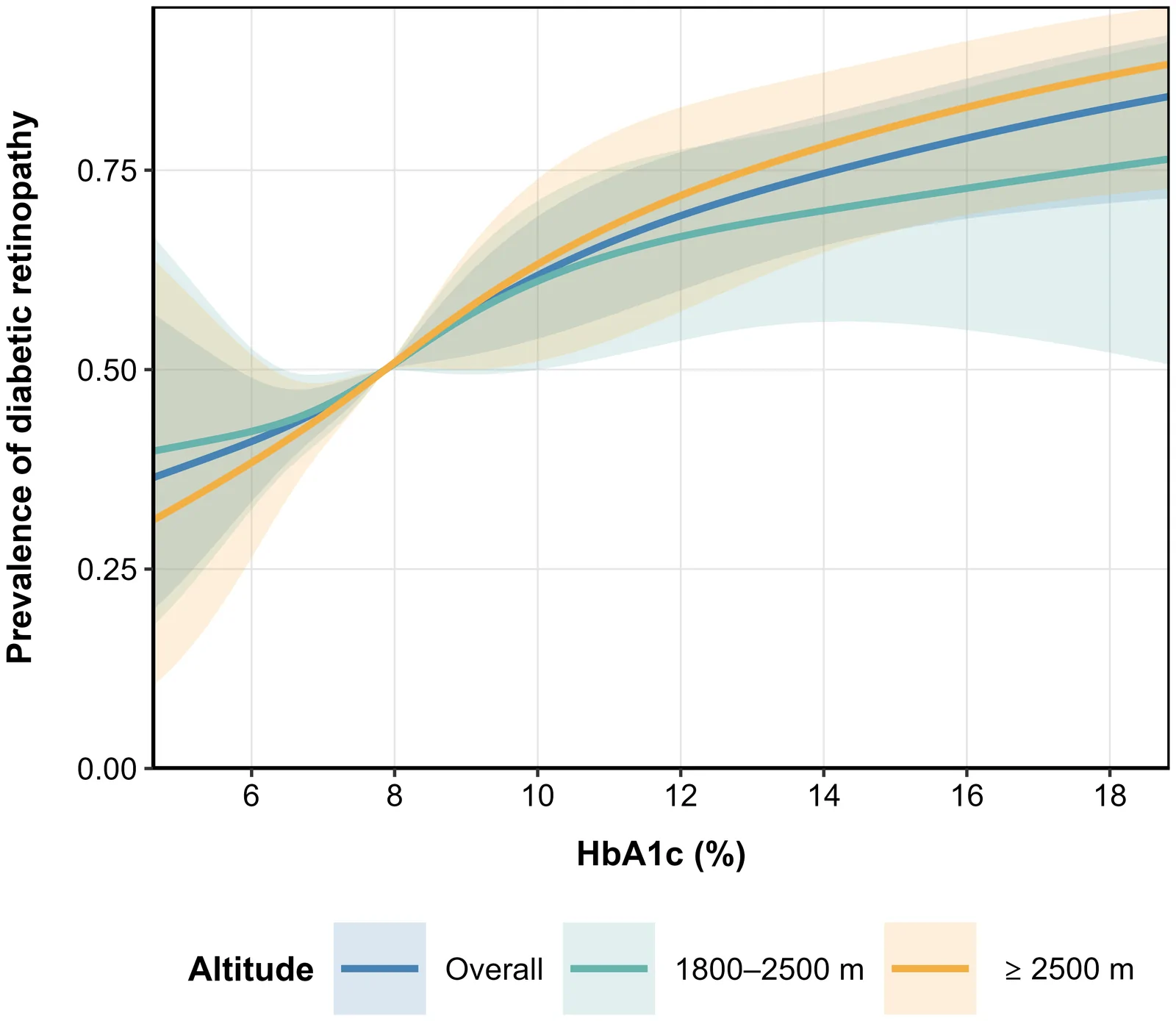
Restricted cubic spline analysis of the association between HbA1c and the prevalence of DR. RCS analysis with 4 knots, adjusted for age, sex, ethnicity, duration, BMI, medication use, smoking status, hypertension, LDL-C, eGFR, and Hb. Colored solid lines represent the overall population and groups stratified by altitude (1800–2500 m and ≥2500 m), as indicated in the legend. Shaded areas represent 95% CIs. No significant non-linearity detected (*P* > 0.68 for all).

After full adjustment, HbA1c remained independently associated with prevalent DR (OR 1.19, 95% CI 1.12-1.25, *P* < 0.001). Stratified by altitude, the point estimate was nominally higher at ≥2500 m (OR 1.23) than 1800–2500 m (OR 1.14), but altitude did not significantly modify the association (*P* for interaction = 0.213) (**Table 3**). These minor discrepancies in point estimates reflect random sampling fluctuation and do not constitute evidence of effect modification. All strata yielded non-significant *P*-values for non-linearity (*P* > 0.05), supporting a linear dose-response relationship between HbA1c and DR prevalence with no identifiable threshold effect (**Figure 2**). Sensitivity analyses adopting alternative residential altitude definitions are summarized in **Supplementary Table 3**. The association between categorical HbA1c groups and DR prevalence across three sequential adjusted models is presented in **Supplementary Table 4**. Additional sensitivity analyses with further adjustment for contemporaneous glucose measurements yielded consistent results and are presented in **Supplementary Table 5**.

**Table 3:** Association of HbA1c and the prevalence of DR by altitude strata.

Altitude (m)	Model 1^a^	Model 2^a^	Model 3^a^	*P* for interaction^b^
Overall	1.14 (1.09, 1.20)^c^	1.19 (1.12, 1.25)^c^	1.19 (1.12, 1.25)^c^	0.213
1800-2500	1.12 (1.05,1.20)^c^	1.15 (1.07, 1.25)^c^	1.14 (1.06, 1.24)^c^	
≥2500	1.17 (1.09,1.25)^c^	1.22 (1.13, 1.33)^c^	1.23 (1.13, 1.33)^c^	

a Model1: unadjusted. Model2: adjusted for duration, age, sex, ethnicity, BMI, medication use, smoking status, hypertension, LDL-C. Model3: adjusted for duration, age, sex, ethnicity, BMI, medication use, smoking status, hypertension, LDL-C, eGFR, Hb. All models were fitted using the same complete-case analytical cohort (n = 1040); participants with missing outcome or covariate data were excluded before analysis.

b Between HbA1c and altitude group in Model3.

c *P* < 0.001 or *P* = 0.001.

**Figure 3:**
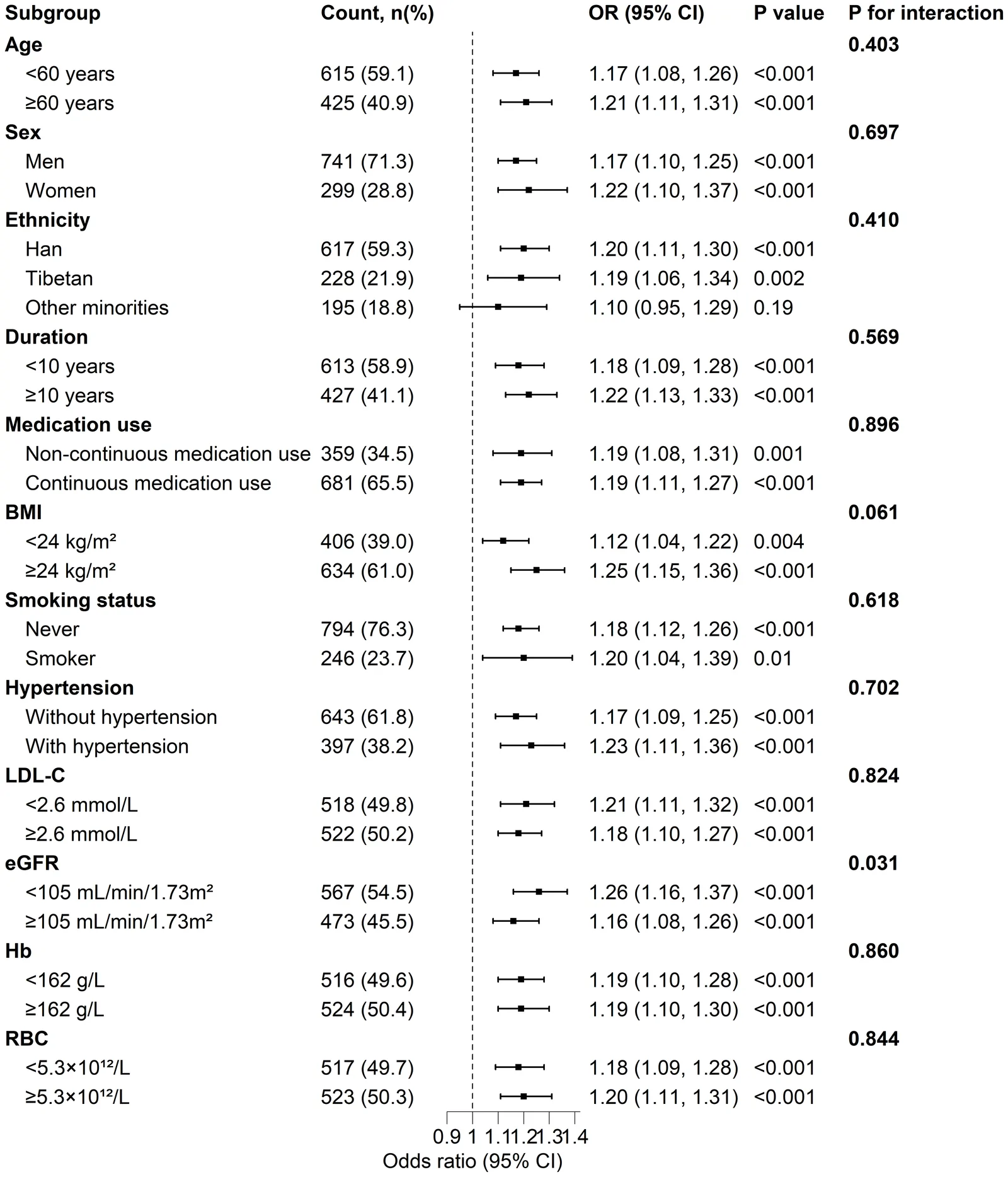
Association between HbA1c and prevalent DR in different subgroups. Each stratified analysis was adjusted for age, sex, ethnicity, duration, BMI, medication use, smoking status, hypertension, LDL-C, eGFR, Hb, and altitude, with the exception of the corresponding stratification variable. Error bars represent 95% CIs.

The association between HbA1c and DR prevalence was generally consistent across prespecified subgroups, with the exception of the “other minorities” subgroup (OR 1.10, 95% CI 0.95-1.29, *P* = 0.190) (**Figure 3**). However, the “other minorities” subgroup included only 195 participants, limiting the statistical power to detect an association. eGFR exhibited a statistically significant interaction (*P* for interaction = 0.031), whereas the interaction by BMI approached but did not reach statistical significance (*P* for interaction = 0.061). The absolute DR prevalence stratified by HbA1c levels and key clinical subgroups is displayed in **Supplementary Table 6**.

**Figure 4:**
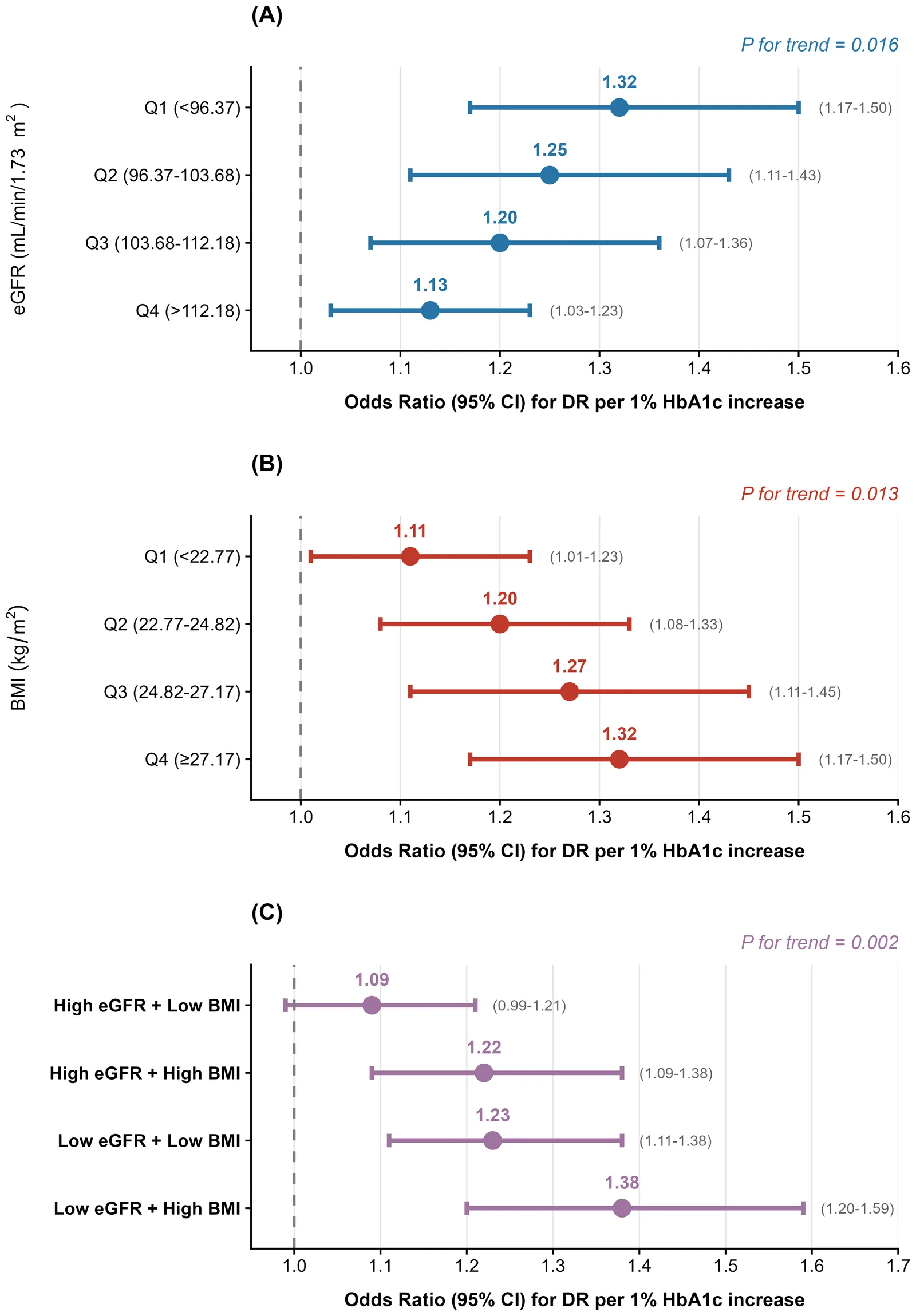
Association between HbA1c and prevalent DR across strata of **(A)** eGFR quartiles, **(B)** BMI quartiles, and **(C)** combined eGFR and BMI risk groups. Each stratified analysis was adjusted for age, sex, ethnicity, duration, BMI, medication use, smoking status, hypertension, LDL-C, eGFR, Hb, and altitude, with the exception of the corresponding stratification variable. In **(C)**, the interaction between eGFR and BMI was not statistically significant (*P* for interaction = 0.715).

To further explore whether the HbA1c-DR association varied linearly across eGFR and BMI levels, we categorized eGFR and BMI into quartiles. For eGFR, the association between HbA1c and DR progressively strengthened across decreasing quartiles. Specifically, the ORs were as follows: Q4: 1.13 (1.03-1.23), Q3: 1.20 (1.07-1.36), Q2: 1.25 (1.11-1.43), and Q1: 1.32 (1.17-1.50) (*P* for trend = 0.016) (**Figure 4A**). For BMI, the association strengthened across increasing quartiles: Q1: 1.11 (1.01-1.23), Q2: 1.20 (1.08-1.33), Q3: 1.27 (1.11-1.45), and Q4: 1.32 (1.17-1.50) (*P* for trend = 0.013) (**Figure 4B**).

In the exploratory combined analysis cross-classifying participants by median values of eGFR and BMI, the HbA1c-DR association was strongest in the low eGFR plus high BMI group (OR 1.38, 95% CI 1.20-1.59), intermediate in the high eGFR plus high BMI (OR 1.22, 95% CI 1.09-1.38) and low eGFR plus low BMI groups (OR 1.23, 95% CI 1.11-1.38), and weakest in the high eGFR plus low BMI group (OR 1.09, 95% CI 0.99-1.21) (*P* for trend = 0.002) (**Figure 4C**). The absence of a significant eGFR×BMI interaction (*P* = 0.715) suggests these modifiers may exert independent rather than synergistic effects, though this warrants confirmation in larger studies.

## 4 Discussion

Our findings suggest that the positive association between HbA1c levels and DR prevalence observed in lowland populations also applies to high-altitude adults with T2DM. Analyses identified that eGFR modified this association, whereas higher BMI showed an exploratory modifying trend reflecting greater disease susceptibility. The highest retinopathy risk related to elevated HbA1c was detected among participants with concurrent lower eGFR and higher BMI.

### 4.1 Mechanisms underlying the stable linear HbA1c-DR association in high-altitude populations

Since the DCCT established a positive association between HbA1c and the risk of diabetic retinopathy onset and progression (6), numerous studies have consistently confirmed this relationship across diverse geographic regions. Recent cross-sectional studies from China demonstrated that each 1% increase in HbA1c was associated with a significantly higher risk of DR, with adjusted OR values of 1.228 (95% CI: 1.010-1.368), 1.269 (95% CI: 1.176-1.369), and 1.237 (95% CI: 1.142-1.341), respectively (26–28). A systematic review and meta-analysis in African populations further reported that poor glycemic control (HbA1c > 7.0%) was associated with increased DR risk (OR = 1.25, 95% CI: 1.14-1.38) (29). In addition, a recent meta-analysis confirmed that elevated HbA1c levels were strongly associated with DR (OR = 2.41, 95% CI: 1.63-3.57) (30). While our findings are broadly consistent with previous evidence, our OR of 1.19 was marginally lower than those reported in prior studies. This could be attributed to the well-documented dissociation between HbA1c and ambient glucose, which was also observed in our high-altitude population: high-altitude residents presented lower plasma glucose with relatively preserved HbA1c levels.

A previous study demonstrated that hypoxia-induced increases in deoxygenated hemoglobin and 2,3-DPG accelerate glycation at equivalent glucose concentrations (31), thereby compromising HbA1c’s validity as a biomarker of chronic glycemia and diluting its predictive value for DR. However, this dissociation does not imply that HbA1c completely loses its ability to reflect chronic glycemic burden. The glycemia-related component retained in HbA1c still represents the cumulative effects of classical hyperglycemia-induced pathogenic pathways. Moreover, the glycohypoxia hypothesis proposes that HbA1c increases hemoglobin oxygen affinity, impairs tissue oxygen unloading, and induces functional hypoxia, thereby activating hypoxia-related signaling pathways such as HIF-1α and contributing to the development of diabetic microvascular complications (16). Under the persistent hypoxic conditions of high altitude, these hypoxia-related effects may be further amplified, particularly in highly oxygen-dependent tissues such as the retina.

### 4.2 Molecular and clinical pathways mediating independent effect modification by eGFR and BMI

Although the overall magnitude of the HbA1c-DR association was relatively modest in the overall high-altitude population, this association was amplified among individuals with higher BMI and lower eGFR. The inconsistent associations between BMI and DR may partly reflect differences in diabetes type, disease duration, metabolic control, and residual β-cell function across study populations. Therefore, BMI may exert a context-dependent influence on DR risk rather than serving as a universal independent risk factor. Higher BMI is characterized by chronic low-grade inflammation, with increased secretion of TNF-α, IL-6, MCP-1 and other adipocytokines (32). Hyperglycemia similarly activates inflammatory pathways through AGE-RAGE signaling and NF-κB activation (33). These overlapping inflammatory cascades may synergistically accelerate retinal endothelial injury and blood-retinal barrier breakdown, thereby strengthening the adverse effect of HbA1c on DR. Obesity is also associated with lipid accumulation, mitochondrial dysfunction and oxidative stress (34). Excess free fatty acids may enhance reactive oxygen species generation, which could potentiate glucose-induced oxidative damage within retinal microvascular endothelial cells (35).

Similarly, renal function also plays an important role in modulating diabetic complications. A Danish nationwide registry-based cohort study found that in populations with diabetes and severe CKD, reduced eGFR merely raised the baseline complication risk but did not modify the association between HbA1c and DR (36). In contrast, significant effect modification by eGFR was evident in our study. Such inconsistent results can be explained by distinct renal profiles of the study populations: our participants mostly had normal-range eGFR with only functional fluctuations, whereas the Danish cohort presented severe CKD accompanied by irreversible glomerular structural atrophy.

Among individuals with largely preserved renal function, a lower eGFR may reflect early systemic microvascular vulnerability. Because the retinal and renal microcirculations share pathological features such as endothelial dysfunction, oxidative stress, inflammation, and impaired autoregulation, lower eGFR may identify individuals whose retinal vasculature is more susceptible to hyperglycemic injury. In advanced CKD, however, microvascular risk may already be substantially elevated through multiple glycemic and nonglycemic pathways, making the incremental effect of HbA1c less apparent and potentially producing a ceiling effect. Altered erythrocyte turnover and anemia may also reduce the reliability of HbA1c in severe CKD. This contrast contextualizes interstudy heterogeneity in renal modification effects. These mechanisms remain speculative because the relevant renal, inflammatory, and endothelial markers were not directly measured.

### 4.3 Biological explanations for absent altitude-driven effect modification

Our study showed that altitude exerted no significant modifying effect on the HbA1c-DR association. Hypoxia accelerates protein glycation to raise HbA1c concentrations yet concurrently promotes erythrocyte glucose uptake and utilization to reduce circulating glycemia. These opposing biological pathways may counterbalance each other, thereby attenuating any modifying influence of altitude on the HbA1c-DR relationship. In addition, although the ≥2500 m subgroup exhibited lower blood glucose levels, the comparable DR prevalence between altitude groups aligns with the limited explanatory power of glycaemic control for microvascular complications. The DCCT/Epidemiology of Diabetes Interventions and Complications (EDIC) Research Group calculated that HbA1c accounted for only up to 11% of the overall variation in the risk of diabetic complications and concluded that most of the remaining variation was associated with factors not captured by glycaemia (37). Thus, other factors, such as altitude-related oxidative stress, hypoxic adaptation, and dietary patterns, may partly explain the observed association between altitude and DR in our study. But without a low-altitude control group, it remains unclear whether altitude could modify the HbA1c-DR association across different altitudinal environments.

The earlier negative result likely stemmed from limited sample size and incomplete adjustment for BMI and renal function confounders. The present study’s larger sample and comprehensive covariate adjustment yield more robust estimates of the true association. These findings also have important implications for clinical practice in high-altitude Qinghai regions, where primary healthcare resources are relatively limited. A tiered risk-based management approach may improve the efficiency of resource allocation. Specifically, standard HbA1c monitoring and glycemic control remain appropriate for the general T2DM population, whereas patients with overweight or obesity and mildly reduced eGFR may warrant closer clinical monitoring and enhanced risk assessment. Importantly, given the cross-sectional observational nature of this study, the above clinical recommendations only support enhanced clinical vigilance and risk stratification for susceptible individuals, rather than confirming causal benefits of BMI- or eGFR-targeted interventions in reducing DR risk. Further prospective and interventional studies are warranted to validate these clinical implications.

Several limitations should be acknowledged. First, the cross-sectional design precludes causal inference and does not allow temporal ordering to be established. Therefore, it remains unclear whether chronic hyperglycemia precedes the onset of DR or whether microvascular injury may, in turn, contribute to disturbances in glycemic homeostasis. Second, this was a single-center retrospective study restricted to residents of Qinghai Province, which may limit the generalizability of the findings to populations living in other high-altitude regions, such as Xizang and western Sichuan. Third, the relatively small number of participants in the ‘other minorities’ subgroup reduced the statistical power of subgroup analyses and may have resulted in imprecise estimates. Fourth, inflammatory biomarkers and oxidative stress mediators, including interleukin-6 and tumor necrosis factor-α, were not measured, preventing mediation analyses of the biological pathways potentially linking hyperglycemia, hypoxic exposure, and retinal microvascular damage. Fifth, several potentially important confounders were unavailable, including antidiabetic medication classes, duration of high-altitude or hypoxic exposure, peripheral or arterial oxygen saturation, dietary patterns, and physical activity levels. In addition, erythropoietin levels were not quantified, which limited our ability to directly assess individual differences in physiological adaptation to chronic hypoxia. Sixth, retinal assessment relied solely on non-widefield fundus photography, without 7-field Early Treatment Diabetic Retinopathy Study (ETDRS) grading or optical coherence tomography (OCT). Consequently, peripheral retinal lesions and some advanced proliferative changes may have been underdetected, potentially leading to underestimation of DR prevalence or severity. In addition, fundus photographs were evaluated by a single ophthalmologist as part of routine clinical care; therefore, inter-grader reliability could not be assessed. Moreover, retinal assessment was based on a single cross-sectional fundus examination without longitudinal follow-up to track DR incidence or progression. Seventh, missing data were handled using complete-case analysis. This approach may have reduced statistical efficiency and introduced selection bias if the data were not missing completely at random. Eighth, this study has several inherent altitude-specific limitations: potential misclassification of individual high-altitude hypoxic exposure, possible measurement bias in HbA1c glycation endpoints under chronic hypoxic hematological adaptation, and the absence of a lowland control group, which restricted our ability to directly quantify altitude-hypoxia-induced modifications on the HbA1c-DR association. In addition, although a previous high-altitude study reported better agreement of the creatinine-based CKD-EPI equation with measured GFR than the Modification of Diet in Renal Disease (MDRD) equation (38), its applicability to the present population remains uncertain because chronic hypoxia may alter hematocrit and serum creatinine concentrations, potentially resulting in residual misclassification of renal function. Ninth, all subgroup and interaction analyses were exploratory, and no formal correction for multiple testing was conducted. Multiple comparisons may inflate type I error, such that several nominally significant subgroup results might arise merely by chance. Tenth, we did not perform sensitivity analyses using alternative exclusion criteria or distinct BMI and eGFR cutoff values. Thus, the robustness of the primary findings to changes in variable definitions and analytic assumptions requires further investigation.

Future research should prioritize prospective longitudinal cohort studies to clarify temporal and causal relationships, together with multicenter collaborative validation across diverse high-altitude regions to assess the reproducibility and generalizability of the present findings. In parallel, preclinical animal models are needed to elucidate the shared molecular pathways through which chronic hypoxia, protein glycation, renal dysfunction, and retinal microvascular injury may interact in the development and progression of DR.

## 5 Conclusions

Our findings provide novel evidence that the positive HbA1c-DR association persists in high-altitude adults with T2DM and is further modulated by eGFR. Higher BMI exhibited an exploratory modulating trend linked to elevated susceptibility to DR. Clinically, apart from routine glycemic management, targeted monitoring and intervention for overweight/obesity and early renal functional fluctuation may facilitate identification of high-risk individuals who stand to benefit from intensive integrated cardiometabolic and retinal care.

## Data Availability

The datasets analyzed in this study are not publicly available but are available from the corresponding author upon reasonable request. Requests to access the datasets should be directed to Yanjun Chen, 504456352@qq.com.

## References

[1] Kramer CK Rodrigues TC Canani LH Gross JL Azevedo MJ . Diabetic retinopathy predicts all-cause mortality and cardiovascular events in both type 1 and 2 diabetes. Diabetes Care. (2011) 34:1238–44. doi: 10.2337/dc11-0079 21525504 PMC3114518

[2] Stratton IM Kohner EM Aldington SJ Turner RC Holman RR Manley SE . UKPDS 50: Risk factors for incidence and progression of retinopathy in type II diabetes over 6 years from diagnosis. Diabetologia. (2001) 44:156–63. doi: 10.1007/s001250051594 11270671

[3] Estacio R McFarling E Biggerstaff S Jeffers B Johnson D Schrier R . Overt albuminuria predicts diabetic retinopathy in Hispanics with NIDDM. Am J Kidney Dis. (1998) 31:947–53. doi: 10.1053/ajkd.1998.v31.pm9631838 9631838

[4] Yau JWY Rogers SL Kawasaki R Lamoureux EL Kowalski JW Bek T . Global prevalence and major risk factors of diabetic retinopathy. Diabetes Care. (2012) 35:556–64. doi: 10.2337/dc11-1909 22301125 PMC3322721

[5] Chew EY Ambrosius WT Davis MD Danis RP Gangaputra S Greven CM . Effects of medical therapies on retinopathy progression in type 2 diabetes. N Engl J Med. (2010) 363:233–44. doi: 10.1056/NEJMoa1001288 20587587 PMC4026164

[6] The Diabetes Control and Complications Trial Research Group . The relationship of glycemic exposure (HbA1c) to the risk of development and progression of retinopathy in the diabetes control and complications trial. Diabetes. (1995) 44:968–83. doi: 10.2337/diab.44.8.968 7622004

[7] Alkhaldy HY Awan ZA Abouzaid AA Elbahaey HM Amoudi SMA Shehata SF . Effect of altitude on hemoglobin and red blood cell indices in adults in different regions of Saudi Arabia. IJGM. (2022) 15:3559–65. doi: 10.2147/IJGM.S358139 35386861 PMC8979750

[8] Martí-Mateos Y Safari Z Bevers S Midha AD Flanigan WR Joshi T . Red blood cells serve as a primary glucose sink to improve glucose tolerance at altitude. Cell Metab. (2026) 38:529–545.e8. doi: 10.1016/j.cmet.2026.01.019 41720104 PMC12923992

[9] Sayarlioglu H Erkoc R Algun E Erem C Atmaca H Dogan E . Nephropathy and retinopathy in type 2 diabetic patients living at moderately high altitude and sea level. Renal Failure. (2005) 27:67–71. doi: 10.1081/JDI-42794 15717637

[10] Maxfield A Hadley C Hruschka DJ . The relationship between altitude and BMI varies across low- and middle-income countries. Am J Hum Biol. (2024) 36:e24036. doi: 10.1002/ajhb.24036 38213006

[11] Ma J Niu H Ma X Han C Qu Y . Effects of long-term high-altitude exposure on retinal and choroidal microcirculation. Graefes Arch Clin Exp Ophthalmol. (2022) 260:3525–32. doi: 10.1007/s00417-022-05699-2 35678838

[12] Ren Q Lv X Yang L Yue J Luo Y Zhou L . Erythrocytosis and performance of HbA1c in detecting diabetes on an oxygen-deficient plateau: A population-based study. J Clin Endocrinol Metab. (2020) 105:dgaa001. doi: 10.1210/clinem/dgaa001 31904080

[13] Ma J Niu H Han C Qu Y . Quantify retinal structure in high-altitude residents with and without high altitude polycythemia. BMC Ophthalmol. (2023) 23:6. doi: 10.1186/s12886-022-02674-7 36597056 PMC9811807

[14] Yin W Ye Y Du R Wang S Zhu H Guo Y . Association of hemoglobin with decreased prevalence of diabetic retinopathy among Tibetan male patients. Sci Rep. (2025) 15:13315. doi: 10.1038/s41598-025-97061-9 40246958 PMC12006545

[15] Han C Zheng X-X Zhang W-F . High altitude retinopathy: An overview and new insights. Travel Med Infect Dis. (2024) 58:102689. doi: 10.1016/j.tmaid.2024.102689 38295966

[16] Akl MM Ahmed A . Glycohypoxia: a hypothesis linking chronic hyperglycemia to functional hypoxia and diabetic complications in type 2 diabetes. Med Gas Res. (2026) 16:454–61. doi: 10.4103/mgr.MEDGASRES-D-25-00137 41830794 PMC13456387

[17] Chen X Yuan W Zeng Y Wu H Pi L Yang Y . Serum cystatin C as a biomarker for diabetic retinopathy and its role in diabetic retinopathy-diabetic kidney disease comorbidity. J Transl Med. (2026) 24:161. doi: 10.1186/s12967-025-07552-6 41527091 PMC12888239

[18] Sabanayagam C Sultana R Banu R Rim T Tham YC Mohan S . Association between body mass index and diabetic retinopathy in Asians: the Asian Eye Epidemiology Consortium (AEEC) study. Br J Ophthalmol. (2022) 106:980–6. doi: 10.1136/bjophthalmol-2020-318208 33622697

[19] Zhou Y Zhang Y Shi K Wang C . Body mass index and risk of diabetic retinopathy: A meta-analysis and systematic review. Med (Baltimore). (2017) 96:e6754. doi: 10.1097/MD.0000000000006754 28562529 PMC5459694

[20] Zhang D Wang S Li M Yan X Sun Z Zhang C . Prevalence and risk factors of diabetic retinopathy in Tibet. Chin J Primary Med Pharm. (2022) 29:835–40. doi: 10.3760/cma.j.issn.1008-6706.2022.06.008 30704229

[21] Zhang X Zhang M Li C Huang Z Yu M Wang L . Associations between glycated hemoglobin and glucose indicators in adults in areas at different altitude in China. Chin J Epidemiol. (2023) 44:401–7. doi: 10.3760/cma.j.cn112338-20220814-00710 36942334

[22] Qinghai Provincial Bureau of Statistics . Qinghai Statistical Yearbook 2024. Available online at: http://tjj.qinghai.gov.cn/nj/2024/indexch.htm (Accessed July 16, 2026).

[23] León-Velarde F Maggiorini M Reeves JT Aldashev A Asmus I Bernardi L . Consensus statement on chronic and subacute high altitude diseases. High Alt Med Biol. (2005) 6:147–57. doi: 10.1089/ham.2005.6.147 16060849

[24] Zheng R Xu Y Li M Wang L Lu J Wang T . Altitudes and hemoglobin A1c values: An analysis based on two nationwide cross-sectional studies. Diabetes Care. (2024) 47:e11–3. doi: 10.2337/dc23-1549 38109429

[25] Fundus Disease Group of Ophthalmological Society of Chinese Medical Association Fundus Disease Group of Ophthalmologist Branch of Chinese Medical Doctor Association . Evidence-based guidelines for diagnosis and treatment of diabetic retinopathy in China (2022). Chin J Ocul Fundus Dis. (2023) 39:99–124. doi: 10.3760/cma.j.cn511434-20230110-00018 30704229

[26] Yang S Liu H Liang Y Wu L Zheng Q Wu J . Association between glycated hemoglobin and diabetic retinopathy in individuals with diabetes: A focus on the modifying effect of ambulatory blood pressure. CIA. (2025) 20:1029–38. doi: 10.2147/CIA.S514622 40687343 PMC12275907

[27] Tan H Wang X Ye K Lin J Song E Gong L . Prevalence and risk factors of diabetic retinopathy among Chinese adults with type 2 diabetes in a suburb of Shanghai, China. PloS One. (2022) 17:e0275617. doi: 10.1371/journal.pone.0275617 36194621 PMC9531829

[28] Cui Y Zhang M Zhang L Zhang L Kuang J Zhang G . Prevalence and risk factors for diabetic retinopathy in a cross-sectional population-based study from rural southern China: Dongguan Eye Study. BMJ Open. (2019) 9:e023586. doi: 10.1136/bmjopen-2018-023586 31530585 PMC6756414

[29] Shiferaw WS Akalu TY Desta M Kassie AM Petrucka PM Assefa HK . Glycated hemoglobin A1C level and the risk of diabetic retinopathy in Africa: A systematic review and meta-analysis. Diabetes Metab Syndrome: Clin Res Rev. (2020) 14:1941–9. doi: 10.1016/j.dsx.2020.10.003 33039936

[30] Alarbash AH Almutairi SN Alazmi HR Almutairi A Almutairi A Alarbash AH . The contributions of glycated hemoglobin (HbA1c), triglycerides, and hypertension to diabetic retinopathy: Insights from a meta-analysis. Cureus. (2025) 17:e79066. doi: 10.7759/cureus.79066 40109786 PMC11920856

[31] Smith RJ Koenig RJ Binnerts A Soeldner JS Aoki TT . Regulation of hemoglobin AIc formation in human erythrocytes *in vitro*. Effects of physiologic factors other than glucose. J Clin Invest. (1982) 69:1164–8. doi: 10.1172/JCI110552 7068852 PMC370181

[32] Donath MY Drucker DJ . Obesity, diabetes, and inflammation: Pathophysiology and clinical implications. Immunity. (2025) 58:2373–82. doi: 10.1016/j.immuni.2025.09.011 41045922

[33] Srivastava R Mishra D Singh D Reddy MY Parihar S Palandurkar K . The AGE-RAGE-oxidative stress axis: A paradigm shift in understanding diabetic retinopathy. Indian J Ophthalmol. (2026) 74:190–5. doi: 10.4103/IJO.IJO_1643_25 41581033 PMC12947087

[34] Choi W Woo GH Kwon T-H Jeon J-H . Obesity-driven metabolic disorders: The interplay of inflammation and mitochondrial dysfunction. Int J Mol Sci. (2025) 26:9715. doi: 10.3390/ijms26199715 41096980 PMC12525337

[35] Toma C Ferdeghini D Ola Pour MM Venkatesan S De Cillà S Grossini E . Oxidative stress in diabetic retinopathy: Pathogenic mechanisms, biomarkers and clinical implications. Antioxidants. (2026) 15:425. doi: 10.3390/antiox15040425 42072067 PMC13113219

[36] Kofod DH Carlson N Almdal TP Bomholt T Torp-Pedersen C Nørgaard K . The association between hemoglobin A1c and complications among individuals with diabetes and severe chronic kidney disease. Diabetes Care. (2025) 48:1400–9. doi: 10.2337/dc25-0339 40481664 PMC12281975

[37] Lachin JM Genuth S Nathan DM Zinman B Rutledge BN DCCT/EDIC Research Group . Effect of glycemic exposure on the risk of microvascular complications in the diabetes control and complications trial--revisited. Diabetes. (2008) 57:995–1001. doi: 10.2337/db07-1618 18223010

[38] Zhang M Cui J Xiao L . Evaluation of the accuracy of four commonly used formulae on glomerular filtration rate in military officers and soldiers who stationed at high altitude with chronic kidney disease. Med J Natl Defending Forces Northwest China. (2021) 42:313–8. doi: 10.16021/j.cnki.1007-8622.2021.05.010

