# Supplementary material for: Association between glycated hemoglobin A1c and diabetic retinopathy in adults with type 2 diabetes mellitus residing at high altitudes
Source: Front Endocrinol (Lausanne). 2026 Aug 27;17:1889928. doi: 10.3389/fendo.2026.1889928 (PMC13557970; doi:10.3389/fendo.2026.1889928)
Supplement: Supplementary file 1 [file Table1.docx]

**Supplementary Table 1. Distribution of DR severity among study participants.**

| **DR severity** | **n (%)** |
| --- | --- |
| No DR | 763 |
| Mild NPDR (stage I) | 124 |
| Moderate NPDR (stage II) | 104 |
| Severe NPDR (stage III) | 44 |
| PDR (stages IV-VI) | 5 |

Abbreviations: NPDR, non-proliferative diabetic retinopathy; PDR, proliferative diabetic retinopathy.

**Supplementary Table 2. Comparison of baseline characteristics between included participants and a representative sample of excluded participants.**

| Characteristics at Baseline | Total | Excluded | Included | *P*-value |
| --- | --- | --- | --- | --- |
| No | 1280 | 240 | 1040 |  |
| Age, years | 57.0 (51.0, 63.0) | 56.0 (50.0, 62.3) | 58.0 (51.0, 63.0) | 0.056 |
| Women, n (%) | 369 (28.8) | 70 (29.2) | 299 (28.8) | 0.961 |
| Ethnicity, n(%) |  |  |  | 0.056 |
| Han | 752 (58.8) | 135 (56.3) | 617 (59.3) |  |
| Tibetan | 231 (18.1) | 36 (15.0) | 195 (18.8) |  |
| Other minorities | 297 (23.2) | 69 (28.8) | 228 (21.9) |  |
| Duration, years | 7.0 (4.0, 13.0) | 6.0 (1.0, 12.3) | 8.0 (4.0, 13.0) | <0.001 |
| Non-continuous medication use, n (%) | 464 (36.3) | 105 (43.8) | 359 (34.5) | 0.009 |
| Altitude, m | 2250.0 (2250.0, 2848.0) | 2250.0 (2250.0, 2850.0) | 2250.0 (2250.0, 2848.0) | 0.807 |
| BMI, kg/m^2^ | 24.80 (22.72, 27.17) | 24.64 (22.40, 27.17) | 24.82 (22.77, 27.17) | 0.233 |
| Hypertension, n(%) | 483 (37.7) | 86 (35.8) | 397 (38.2) | 0.548 |
| SBP, mmHg | 126.0 (114.0, 140.0) | 125.5 (112.0, 139.0) | 127.0 (115.0, 140.0) | 0.345 |
| DBP, mmHg | 78.0 (70.0, 86.0) | 77.0 (69.8, 85.0) | 78.0 (71.0, 86.0) | 0.244 |
| Smoker, n(%) | 292 (22.8) | 46 (19.2) | 246 (23.7) | 0.159 |
| DR, n (%) | 321 (25.5) | 44 (19.9) | 277 (26.6) | 0.046 |
| HbA1c, % | 7.9 (6.5, 9.8) | 8.5 (6.4, 10.0) | 7.9 (6.5, 9.7) | 0.304 |
| FPG, mmol/L | 7.36 (5.96, 9.19) | 7.48 (5.91, 9.18) | 7.30 (5.96, 9.19) | 0.703 |
| 2hPG, mmol/L | 14.59 (11.46, 17.49) | 14.83 (11.65, 17.49) | 14.50 (11.43, 17.49) | 0.493 |
| AUC_G_, mmol·h/L | 37.45 (29.91, 45.28) | 39.53 (32.24, 50.29) | 37.03 (29.50, 44.87) | <0.001 |
| TG, mmol/L | 1.80 (1.24, 2.76) | 1.60 (1.18, 2.48) | 1.85 (1.26, 2.83) | 0.007 |
| HDL-C, mmol/L | 1.08 (0.93, 1.27) | 1.10 (0.95, 1.28) | 1.07 (0.93, 1.27) | 0.117 |
| LDL-C, mmol/L | 2.61 (2.15, 3.16) | 2.64 (2.01, 3.23) | 2.60 (2.17, 3.15) | 0.883 |
| TC, mmol/L | 4.66 (4.00, 5.40) | 4.72 (4.05, 5.40) | 4.64 (4.00, 5.40) | 0.579 |
| Scr, μmol/L | 59.25 (50.60, 68.90) | 61.80 (51.47, 70.57) | 58.50 (50.27, 68.30) | 0.003 |
| eGFR, mL/min/1.73 m² | 103.49 (96.01, 112.03) | 102.55 (94.16, 111.82) | 103.68 (96.37, 112.18) | 0.097 |
| RBC, ×10^12^/L | 5.28 (4.93, 5.66) | 5.18 (4.82, 5.55) | 5.30 (4.94, 5.69) | <0.001 |
| Hb, g/L | 161.0 (149.0, 172.0) | 160.0 (146.0, 169.0) | 162.0 (150.0, 173.0) | 0.014 |

**Supplementary Table 3. Sensitivity analyses of the association between HbA1c and diabetic retinopathy using alternative definitions of residential altitude.**

| **Sensitivity analysis** | **HbA1c OR (95% CI)** | ***P*-value** | ***P* for interaction** |
| --- | --- | --- | --- |
| Continuous altitude | 1.18 (1.12-1.25) | <0.001 | 0.541 |
| 2400 m cutoff | 1.20 (1.11-1.29) | <0.001 | 0.543 |
| 2600 m cutoff | 1.20 (1.11-1.29) | <0.001 | 0.475 |

Values are presented as odds ratios (ORs) and 95% confidence intervals (CIs). Logistic regression models were used to assess the association between HbA1c and diabetic retinopathy (DR). In the continuous-altitude model, residential altitude was modeled per 500-m increase, and an interaction term between HbA1c and continuous altitude was included. In the alternative cutoff analyses, altitude strata were redefined using 2400 m and 2600 m cutoffs, respectively, and interaction terms between HbA1c and altitude group were tested. All models were adjusted for age, sex, ethnicity, duration, BMI, medication use, smoking status, hypertension, LDL-C, eGFR, and Hb.

**Supplementary Table 4. Association of HbA1c categories with the prevalence of diabetic retinopathy.**

| **HbA1c category** | **Model 1^a^** | **Model 2^a^** | **Model 3^a^** | ***P* for trend^b^** |
| --- | --- | --- | --- | --- |
| < 7.0% | Reference | Reference | Reference | <0.001 |
| 7.0–8.9% | 2.09 (1.46, 3.02)**^c^** | 2.10 (1.44, 3.07)**^c^** | 2.13 (1.46, 3.13)**^c^** |  |
| ≥ 9.0% | 2.66 (1.87, 3.82)**^c^** | 3.39 (2.32, 5.01)**^c^** | 3.45 (2.35, 5.12)**^c^** |  |

^a^ Model 1: unadjusted. Model 2: adjusted for duration, age, sex, ethnicity, BMI, medication use, smoking status, hypertension, LDL-C. Model 3: adjusted for age, sex, ethnicity, duration, BMI, medication use, smoking status, hypertension, LDL-C, eGFR, Hb, and altitude.

^b^ *P* for trend was calculated by modeling HbA1c categories as an ordinal variable (scores 1-3) in Model 3.

^c^ *P*<0.001.

**Supplementary Table 5. Sensitivity analyses of the association between HbA1c and prevalent DR after additional adjustment for contemporaneous glucose measurements.**

| **Model** | **OR (95% CI)** | ***P*-value** |
| --- | --- | --- |
| Model 3 | 1.19 (1.12-1.26) | <0.001 |
| Model 3+FPG | 1.17 (1.10-1.25) | <0.001 |
| Model 3+2hPG | 1.19 (1.12-1.27) | <0.001 |
| Model 3+AUC_G_ | 1.20 (1.12-1.28) | <0.001 |

Model 3 was adjusted for age, sex, ethnicity, duration, BMI, medication use, smoking status, hypertension, LDL-C, eGFR, Hb, and altitude. Sensitivity analyses were performed by additionally adjusting Model 3 for FPG, 2hPG, or AUC_G_ separately. ORs represent the association between HbA1c (per 1% increase) and prevalent DR.

**Supplementary Table 6. Absolute prevalence of diabetic retinopathy across HbA1c categories and key clinical subgroups**

| **Subgroup** | **Category** | **Participants, n** | **DR cases, n** | **DR prevalence (%)** |
| --- | --- | --- | --- | --- |
| HbA1c | <7% | 357 | 59 | 16.5 |
|  | 7–<9% | 338 | 99 | 29.3 |
|  | ≥9% | 345 | 119 | 34.5 |
| Age | <60 years | 615 | 130 | 21.1 |
|  | ≥60 years | 425 | 147 | 34.6 |
| Sex | Men | 741 | 200 | 27 |
|  | Women | 299 | 77 | 25.8 |
| Ethnicity | Han | 617 | 176 | 28.5 |
|  | Tibetan | 228 | 54 | 23.7 |
|  | Other minorities | 195 | 47 | 24.1 |
| Duration | <10 years | 613 | 77 | 12.6 |
|  | ≥10 years | 427 | 200 | 46.8 |
| Medication use | Non-continuous medication use | 359 | 71 | 19.8 |
|  | Continuous medication use | 681 | 206 | 30.2 |
| BMI | <24 kg/m^2^ | 406 | 110 | 27.1 |
|  | ≥24 kg/m^2^ | 634 | 167 | 26.3 |
| Smoking status | Never | 794 | 219 | 27.6 |
|  | Smoker | 246 | 58 | 23.6 |
| Hypertension | Without hypertension | 643 | 133 | 20.7 |
|  | With hypertension | 397 | 144 | 36.3 |
| LDL-C | <2.6 mmol/L | 518 | 133 | 25.7 |
|  | ≥2.6 mmol/L | 522 | 144 | 27.6 |
| eGFR | <105 mL/min/1.73 m² | 567 | 177 | 31.2 |
|  | ≥105 mL/min/1.73 m² | 473 | 100 | 21.1 |
| Hb | <162 g/L | 516 | 139 | 26.9 |
|  | ≥162 g/L | 524 | 138 | 26.3 |
| RBC | <5.3×10^12^/L | 517 | 147 | 28.4 |
|  | ≥5.3×10^12^/L | 523 | 130 | 24.9 |
